# *Divinum opus est sedare dolorem*: the power (and beauty) of early palliative care

**DOI:** 10.1093/oncolo/oyae348

**Published:** 2024-12-09

**Authors:** Elena Bandieri, Leonardo Potenza, Eduardo Bruera, Mario Luppi

**Affiliations:** Oncology and Palliative Care Units, Civil Hospital Carpi, Local Health Agency (USL), Modena 41012, Italy; Hematology Unit and Chair, Azienda Ospedaliera Universitaria di Modena and Department of Medical and Surgical Sciences, University of Modena and Reggio Emilia, Modena 41124, Italy; Palliative Care & Rehabilitation Medicine, UT M.D. Anderson Cancer Center, Houston, TX 77030, United States; Hematology Unit and Chair, Azienda Ospedaliera Universitaria di Modena and Department of Medical and Surgical Sciences, University of Modena and Reggio Emilia, Modena 41124, Italy

*“How beautiful the world is! But why does this give a sense of pain?.”*

Boris Pasternak, Doctor Zhivago

Mrs A., at the age of 58 years, was referred by her oncologist to the early palliative care (EPC) outpatient ambulatory clinic of our cancer center in Modena, because of breast cancer progression in the lung and bones.^1,2^ She had been diagnosed with advanced breast cancer 3 years earlier, with bone and lymph node metastases, and had completed 5 lines of cancer therapy. Our initial questions to verify her level of awareness of the diagnosis demonstrated her clear understanding. Mrs A. was accompanied by her partner Mr M., who worriedly questioned everything that was said and was even more worried about the silences between the words.

I (E.B.) remember that first meeting with Mrs. A very well. She carried herself with a great dignity and strength, albeit veiled somewhat by fragility. Mrs. A. was an expert in literature and passionate about art. The phrase with which she began the conversation was a quote from the novel *Doctor Zhivago*^3^: “Dear doctor, how beautiful the world is! But why does this give a sense of pain?.” “But… you see, doctor”—she added—“beyond the quotes that I love. My greatest pain now—I would say unbearable—is related to the bone metastases that no longer allow me to live, and sometimes not even to be able to think about continuing to live. I came here hoping for a quick end to this terrible pain that tears my body and soul apart.” From that moment, I assured Mrs. A. that we would work to find a solution for her pain.

The following meeting took place without Mr. M., so that we could talk freely about the topic that was closest to her heart: “My need is to be able to talk about my death, to be able to prepare myself in some way. Just being able to communicate...I think...can help me and make me feel better.” “I have already faced other situations of loss and suffering in my life, many years ago. The fear of a fatal disease is ancient for me, and this is why I immediately thought of euthanasia.” “I’ve tried to live with it for years, but... now... the weight of this baggage is too heavy for me alone. Can I share it with you, doctor?,” hinting at a smile.

Over the course of 14 months, during repeated clinic visits, conversations about the suffering caused by the inexpressible thought of death transformed into reflections on the great passion of Mrs. A.’s life: Renaissance art, in particular works by Piero della Francesca. A few months before her death, I asked her: “Many months have passed; how is the pain and how has it changed in these months?” The answer was: “The physical pain is well controlled by drugs. The other pain, that of the soul, terrible at first, had forced me to think about death with desperation at every moment and gave me neither respite nor hope. With the first improvement, time after time, and then, the disappearance of the soul pain, I have rediscovered the precious time of the present moment, a space not previously known, which allowed me to live again and to be able to dedicate myself entirely to what I have always loved most: art.”

With a smile, she added: “Thank you also for the space for authentic relationships in this clinic, I have found a heaven that I feel I can rely upon. Thanks to this and to my love for art, I am managing to prepare myself for the most important part of my life.”

Two months before Mrs. A.’s death, by shared decision, the home palliative care service was activated. She died at home with her partner by her side. My relationship with her, based on listening and truth, allowed for a clearer understanding of the utterance of the chorus in Aeschylus’ Agamemnon—“learning through suffering.”^4^

Shortly after Mrs A.‘s death, I set off with some colleagues to Monterchi (a small ancient Italian village on the Tuscan/Umbrian border), with a strong desire to re-expose us to a masterpiece of Italian Renaissance art, the Madonna del Parto, by Piero della Francesca.

**Figure 1.**
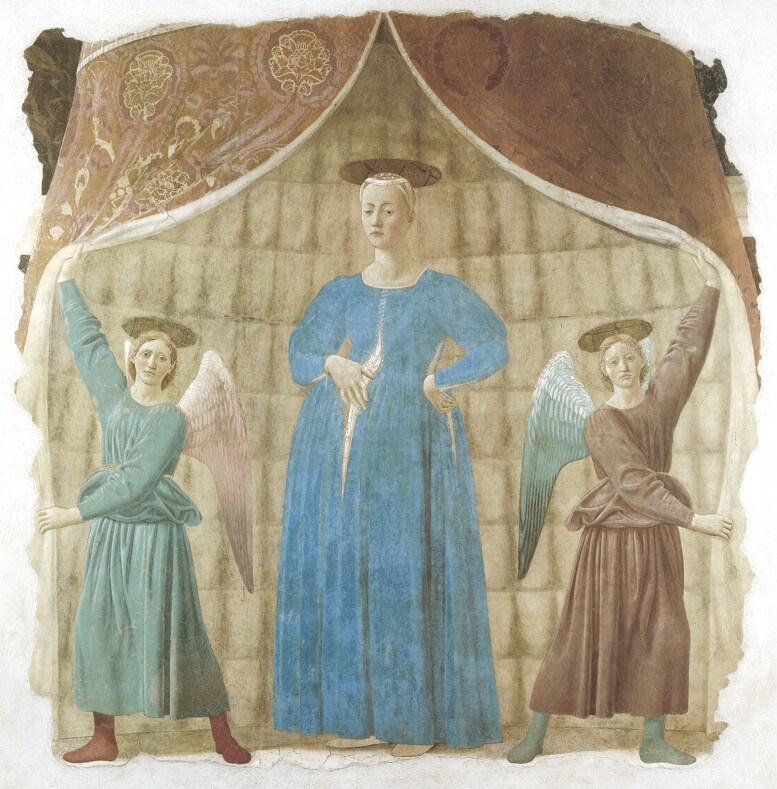
Madonna del Parto, Monterchi, Italy.

The Madonna del Parto was painted in Monterchi, the birthplace of the painter’s mother, between 1455 and 1465^5^ (Figure 1). The pregnant Madonna appears at the center of the scene, with a posture that emanates the fatigue and heaviness of the body: a woman, without divine attributes, who is crossing the threshold of the mystery of birth. The angels are 2 perfectly symmetrical and complementary figures, in shape and colors (the robe and wings of the right angel are respectively green and red, perfectly symmetrical, and complementary to the red robe and the green wings of the angel on the left). The serious, unsmiling gaze of the 2 angels stares directly at the viewer, inviting contemplation of the mystery that is about to be revealed. The light blue fabric is open both on the front and on the left side due to the pregnant belly, showing white under the dress, which looks like a sort of “cut,” or “laceration.” One has the perception that here, Piero intended to refer to the same laceration of the human condition, caught in its vulnerability of birth and death. The gesture of the hand evokes the idea of care that welcomes a new life, and, at the same time, repairs the pain, the “cut” of birth; a “cut” which will, inevitably, also be that of death. The second “‘cut’” on the left side, is also repaired by the other hand, the emblem of care. The royal pavilion delimits a sacred space, in which both the beginning of life and its natural, expected epilogue, are welcomed. The Madonna’s gaze, turned three-quarters, appears lowered, distant from the viewer, as if she wants to retreat into a private and transcendent dimension, with a premonition of death and a dignified awareness of the human condition. The angels’ serious gaze is a sort of ‘“memento mori”‘ (remember that you must die).

Mrs A. loved Simone Weil and I often discussed passages from *The Notebooks of Simone Weil*, with her. If, as Weil suggests, “What is sacred in art is beauty,”^6^ then it is precisely through the language of beauty that Piero inspired and continues to inspire the sense of the sacred connected to the condition of being human, recognizable in its indissoluble superposition of Life, Death, Body, and Soul. And above all, at least to us, the Madonna del Parto inspires the idea of care as a human and medical response to life and suffering.

The Hippocratic principle “*Divinum opus est sedare Dolorem*” (to alleviate pain is divine work) represents the first medical obligation—to alleviate suffering, while also indicating that its fulfillment has an inherent, transcendent value. In more recent times, the Heideggerian philosophical conceptualization of care has introduced a holistic dimension into medicine, in which the person is at the center and is hinged in the frame of a human relationship.^7^ Medical care, particularly palliative care, is based on the belief that, in the face of suffering from an incurable disease, sensitive communication, based on trust and collaboration, can improve the situation, and reaffirm that life is worth living. Without cultivating this deep and meaningful component of care (which may be called *compassionate care*), the physician runs the risk of becoming a mere *provider of services* and losing a more complex humanistic vision.

In the case of Mrs A., cancer pain resulted from a combination of physical and spiritual sufferings, caused by the disease, oncologic treatments, and her thoughts and feelings. E.B. and the healthcare team did not underestimate the multidimensional origin of cancer pain and grew a spiritual connection with the patient and her caregiver, by anticipating the palliative care intervention, as soon as possible. The early palliative care (EPC) intervention allowed participatory listening and ensured presence: the narration of suffering and its recognition “by the other” “with the other” helped the patient to satisfy the need to unify a body-mind vision, that had been fragmented by medical treatments.

Anticipated palliative care interventions, that is, EPC have been recognized as an effective response to the suffering of patients with cancer and their primary caregivers.^8,9^ The practice of EPC allows the necessary time to develop and nurture the central role of the physician-patient relationship, renewing the ancient alliance that grounds the Hippocratic medical oath and broadening the horizons of medical action beyond technology and drugs. In our experience, EPC also offers an alternative to practices of therapeutic obstinacy and medical futility, instead of regarding death as part of life.^10^ Multiple barriers exist to integrate palliative medicine in cancer care.^11^ The recent experience of the Community of Practice platform, launched by ASCO, has made notable advances to meet the multiple critical needs of the palliative oncology community.^11^

We consider the act of palliative caring to be a kind of human relationship, an ethical act that affirms the value of life and harmonizes its appearance (birth) and its fulfillment (death), by allowing those who die to leave a testimony of meaning for those who remain.
